# Parasocial interactions and parasocial relationships on Instagram: An in-depth analysis of fashion and beauty influencers

**DOI:** 10.1016/j.heliyon.2024.e39708

**Published:** 2024-10-22

**Authors:** Xiaoxiao Zhou, Yi Huang, Yuki Inoue

**Affiliations:** a Department of Innovation Science, School of Environment and Society, Institute of Science Tokyo, Tokyo, Japan; b Graduate School of Humanities and Social Sciences, Hiroshima University, Hiroshima, Japan; c Department of Industrial Engineering and Economics, School of Engineering, Institute of Science Tokyo, Tokyo, Japan

**Keywords:** Parasocial relationships strength, Parasocial interaction, Value of influencer’s post, Influencers, Category of posts

## Abstract

Influencer marketing on social media platforms has garnered considerable attention in recent years. This study focuses on beauty and fashion influencers on Instagram and examines how changes in the category of influencer posts, value of influencer posts, and levels of parasocial interaction (PSI) correlate with parasocial relationships (PSRs). A regression analysis of data from 215 influencers and 7285 posts in the Japanese market revealed that enhancing PSIs between influencers and followers is associated with stronger PSRs with followers. An increase in the value of an influencer’s posts was also correlated with stronger PSRs. Furthermore, the interaction between an increase in the value of an influencer’s posts and an increase in PSIs was significantly associated with stronger PSRs. Moreover, when PSIs intensify, an increase in posts that directly promote products, clothing coordination, and influencers’ style introductions is positively and significantly correlated with the strength of PSRs between influencers and followers. Influencers can use these insights to develop strategies for effectively building stronger PSRs.

## 1 Introduction

Social networking service (SNS) create opportunities to expand connections between individuals and fulfill consumers’ need for belonging [1,2]. This has fostered trust in the information shared on social media platforms [3], having a notable impact on consumer behavior [3]. This study emphasizes the important role of social media in shaping consumers’ purchase desires and choices [[4], [5], [6], [7], [8], [9], [10]], rendering it an essential modern marketing tool [6]. Social media influencers, defined as content generators with domain expertise who shape followers’ attitudes and purchase decisions [11], play an important role in marketing strategies [6].

These influencers are opinion leaders in their fields. They can guide followers in their purchasing decisions by strengthening PSRs [12]. This enduring, friendship-like bond boosts followers' purchasing intent [6,13]. Several studies have demonstrated that PSRs affect consumer behavior [[14], [15], [16], [17]], with some examining their influence on consumers’ willingness to pay [6,[18], [19], [20]]. Makmor et al. [14] found that PSRs contribute positively to impulse buying on live-shopping sites. Nadroo et al. [15] found that PSRs are associated with word-of-mouth effects. This enhanced word-of-mouth effect not only increases brand awareness but also leads to more sales opportunities.

Therefore, providing influencers with insights on how to strengthen PSRs is important. Parasocial interaction (PSI) plays an important role in strengthen PSRs, serving as the mechanism through which these relationships are formed and strengthened [18,20]. PSI refers to one-sided relationships in which followers feel an intimate bond with influencers despite no direct interaction, making them feel connected and engaged with the influencers [18,20]. Specifically, direct interaction (e.g., responding to personal comments on social media) is a form of PSI that enhances PSR.This engagement provides followers with a sense of personal connection and recognition from the influencer, which strengthens their relationship. Studies by Djafarova and Rushworth [18] and Lou and Kim [20] investigated the influence of influencer-follower PSIs on the construction of PSRs. Sokolova and Kefi [6] highlighted how influencer-follower PSIs foster PSRs, demonstrating that followers’ trust in influencers is linked to their purchase intentions. Similarly, Huertas and Marine-Roig [21] suggested that social media PSIs contribute significantly to building user relationships, thereby shaping PSRs. Moreover, engaging directly with followers through personalized interactions not only fosters trust but also deepens perceived intimacy and commitment, enhancing the durability and strength of PSRs.

However, PSIs’ effectiveness in enhancing PSRs may vary significantly depending on the specific context in which they are deployed. For example, the influencer content plays an important role in the formation and strengthening of PSRs. High-quality content that resonates with followers can deepen their emotional engagement and loyalty [12,[22], [23], [24], [25], [26]]. When content is perceived as valuable, it attracts sustained attention and interactions from followers. Additionally, the effectiveness of an influencer’s PSI may vary depending on the content category, as different content categories appeal to different audience groups [27]. So, influencers can strengthen their PSR by creating content that truly resonates with followers. Understanding the content-specific factors related with PSI dynamics is important for influencers to improve PSR across different follower groups.

Recent changes in consumer behavior and advancements in social media platform technology have highlighted the importance of understanding the dynamics of influencers and their followers. Over the past decade, the highly interactive and visual content-focused nature of social media platforms such as Instagram and TikTok has introduced new dynamics and challenges for influencer marketing [28]. Social media platforms have enhanced features that facilitate PSIs such as live streaming, interactive stories, and direct messaging. Social media influencer marketing research began to flourish ten years after the concept was introduced in 2008 [29]. The rapid growth of social media influencer marketing research seems to align with the growth of highly interactive and visual content-focused social media [28]. However, previous research has not fully explored how to quantify the value of this content, content categories, and their impacts on PSRs. As Keller noted, consumers increasingly seek personalized content that resonates deeply with them [30,31]. While promoting PSRs through PSIs, influencers increasingly need to consider consumer demands and strategically use PSIs to strengthen PSRs. This includes assessing the value of content for consumers and identifying target consumer demographics for different content categories. Depending on these factors, different effort intensities may be required to enhance PSRs. This study systematically analyzes the dynamics of content value, content category, and PSI on Instagram and their impact on PSR strength. By exploring in depth how these factors work in concert to shape and develop PSR, this study addresses a critical gap in the social media influencer marketing literature and spearheads research in this area, providing brands and influencers with timely theoretical underpinnings and practical guidelines on how to utilize content strategy more effectively.

This study focuses on Japanese fashion and beauty influencers as research subjects because Japan is a key player in the global fashion and beauty markets, with a significant market scale and influence [32,33]. We extract data from influencers’ Instagram posts to elucidate the correlations between changes in influencer-follower PSIs, the value of influencers’ posts, and post categories on the dynamics of PSRs’ strength. Specifically, we examine how changes in the value of influencers’ posts and post proportions in different categories are associated with PSRs’ strength through influencer-follower PSIs. We contribute to the literature by providing a detailed understanding of how these factors collectively relate to PSRs’ strength, thereby offering practical insights for researchers and practitioners in the field of influencer marketing.

## 2 Related works

### 2.1 Influencer

User-generated content on social media platforms has increased rapidly. Users have become active content generators, actively sharing personal stories and reviews of products or services on social media [13,34]. User-generated content (UGC) enables brands to interact and build relationships with their customers [35]. In this context, individuals who have considerable influence over the general user base and influence the purchasing behavior and intentions of their followers are known as influencers [13,20,36]. Influencers predominantly operate in domains such as health and fitness, fashion and beauty, food, and advanced technology [[36], [37], [38]].

Traditionally, celebrity popularity has been primarily driven by activities outside social media, such as television, music, and sports [18,39]. Conversely, influencers distinguish themselves from traditional media celebrities by gaining popularity through the creation of valuable posts and building reciprocal relationships with followers via social media [20,34]. This approach enables influencers to develop closer ties with their followers, thereby fostering trust and credibility [18]. As a result, social media users tend to view their relationships with influencers as friendships rather than just fan relationships.

Influencers’ impact is evident in the content they share with followers—textual and visual narratives about personal lives, lifestyles, and choices—that stimulates consumer emulation [40]. Lou and Yuan [41] contended that followers perceive influencers as friends who share common interests. Dao et al. [42] stated that individuals tend to share their personal experiences and trust information with their close friends or family members. A strong connection between the information sender and receiver enhances the influence of information, rendering the receiver more receptive [42,43]. The influencers’ actions are recognized as beneficial by their followers, thereby offering opportunities to discover new brands and products. Consequently, followers might buy the same products as influencers or suggest them to others [42].

Unlike traditional celebrities, influencers, who are often ordinary individuals rather than entertainers or renowned personalities, cultivate more equitable relationships with their followers [34]. Djafarova and Rushworth [18] revealed that social media influencers are more intimately connected with expertise and purchasing behaviors and foster greater trust and reliability among consumers than conventional celebrities. Their content was considered trustworthy [18,44]. Currently, many influencers act as opinion leaders by providing reliable information, advice, and expertise on products and services [6,13,45,46].

### 2.2 PSRs in the context of social media influencers

PSRs are defined as one-sided emotional and social connections that individuals establish through media figures, such as celebrities or influencers [47]. Rooted in the human instinct for social connection, PSRs allow viewers to establish emotional connections not only through face-to-face PSIs but also through PSIs with individuals on social media [48].

Social media influencers’ PSRs present a unique dynamic compared to traditional media personalities such as television actors. Social media has changed the traditional PSR paradigm by enabling two-way communication [47,48]. The PSI is inherently more interactive on social media because it is two-way.

Second, social media influencers share their lives more frequently through photography, videos, and stories. This constant exposure allows followers to feel close and personally connected to the influencer in the same way as face-to-face intimacy would normally occur. Followers can directly interact with influencers through comments, likes, and shares and potentially receive a response from the influencer [49]. PSI increases intimacy and makes relationships appear more reciprocal than traditional celebrity relationships that are often inaccessible to the average viewer.

Despite this potential, the size of a popular influencer’s fan base implies that not all PSIs are reciprocal, retaining the typical one-way nature of PSR. Understandably, influencers, in the same way as traditional celebrities, are unlikely to respond to every comment or message, creating a seemingly interactive but often one-sided relationship [34,50,51]. Empirical evidence indicates that consumers are becoming increasingly attuned to the relationship between brands and influencers [52,53]. When the content has excess advertising, customers may develop negative emotions. This can damage PSRs with influencers [52]. In these contexts, influencers are often seen as prioritizing brand interests over consumer benefits [54].

Overall, many studies have found that followers identify the relationship between influencers and brands, leading to negative emotions [54]. However, several studies have demonstrated that social media’s effectiveness in stimulating follower intentions depends on the influencers’ ability to establish PSRs with their followers. This capability has been highlighted in several studies [6,55,56]. Research on PSRs has explored the factors influencing these relationships or interactions from various perspectives, including the influencers’ traits, follower characteristics, and the quality of influencer-produced content. Emotionally engaged content can significantly enhance PSRs’ strength. The research findings are summarized in Table 1.

**Table 1:** Summary of influential factors for PSR dynamics.

	Result	Authors
Influencers’ characteristics	Men tend to form PSRs with netizens who contribute to society. Women are more likely to build PSRs with reliable influencers.	Kim and Kim [57]
	Credibility factors such as exposure, credibility, and attractiveness are associated with PSRs.	Sokolova and Kefi [6]
	Influencer’s perceived usability influences PSRs with followers.	Quelhas-Brito et al. [58]
	Attractiveness and influencers’ expertise are key factors in PSRs with followers.	Aw and Chuah [59]
Followers’ characteristics	Followers’ perceived attractiveness of influencers, similarity to influencers, procedural fairness, and interpersonal fairness of their PSIs with influencers are positively related to the strength of their PSRs with influencers.	Yuan and Lou [60]
		Sokolova and Kefi [6]
	Followers’ attachment contributes to the emotional bond between influencers and consumers.	Ki and Kim [19]
		Yuan and Lou [60]
Contents	Content characteristics (design, technology quality, and creativity) are associated with PSRs	Cheung et al. [22]
	Information qualities are associated with PSRs.	Lee and Hong [23]
	Design quality (graphics, audio, and video) is associated with PSRs.	Casaló, Flavián, and Ibáñez-Sánchez [12]
	Emotional narratives are associated with PSRs.	Lim and Lee; Gross, Cui and Wangenheim [24,25]
	Influencers’ self-disclosure is associated with PSRs.	Morikawa [26]

### 2.3 Research gap

Research on the link between content value and PSR strength is still scarce. Although various studies have explored factors related to PSR strength, such as content credibility, information quality, design quality (e.g., graphics, audio, video), technical quality, creativity, emotional narrative, and influencer self-disclosure [12,[22], [23], [24], [25], [26]]. These factors typically work together to enhance the overall content value. However, the existing studies has not thoroughly explored how the perceived content value impacts the for-mation and strengthening of PSRs. Additionally, current research focuses on the role of PSI in PSR strength. Sokolova and Kefi [6] demonstrated how PSI between influencers and followers fosters PSR, showing that followers’ trust in influencers is related to their purchase intentions. Similarly, Huertas and Marine-Roig [21suggested that social media PSIs significantly contribute to shape PSRs. However, the role of content value in shaping PSI strength needs further investigation. Therefore, we further analyzed this aspect and proposed our first research question as follows:

RQ1: “What is the relationship between the value of an influencer’s posts and the strength of PSRs? Additionally, how are fluctuations in the value of an influencer’s posts and levels of PSI collectively associated with the strength of PSRs?”

Furthermore, while the individual characteristics of influencers and followers, as well as content quality, are key elements in enhancing PSRs, changing the individual characteristics of influencers and followers is difficult in the short term. Additionally, not all influencers can quickly create high-quality content despite their clear role in enhancing PSRs. Studies have largely overlooked how strategic content management and interactive engagement with followers can be used to adjust PSR strength. Huertas and Marine-Roig (2016) suggest that social media PSIs contribute significantly to building user relationships, thereby shaping PSRs [21]. Moreover, different content categories have varying effects on audiences. For example, content related to beauty and fitness may relate differently to PSR strength owing to varying audience expectations [36,38]. Given the challenges associated with altering the individual traits of influencers and followers and the variability in influencers’ ability to rapidly produce high-quality content, we must investigate strategic approaches to understand the correlates of content management and interactive engagement in PSRs. Building on our understanding of how different content categories correlate with PSR strength, we further investigated strategic adjustments in PSI strength based on content categories and their potential impacts on improving PSR. Accordingly, we propose the second research question:

RQ2: “What is the relationship between the change in posts’ content categories and the strength of PSRs? How are fluctuations in posts’ content categories and levels of PSIs collectively associated with the strength of PSRs?"

Overall, we elucidate the dynamics among content categories, content value, and the strength of PSIs and their collective correlation with the strength of PSRs. Notably, previous studies have examined these elements mainly from a static perspective, we focus on their changes and interactions over time to gain a deeper understanding of their evolving nature and association with PSRs.

## 3 Methodology

The study gathered data from posts created by influencers on Instagram, a social media platform used to share photos and videos. Instagram was chosen primarily because, unlike text-based platforms such as Twitter, Instagram focuses on visual content, facilitating more vivid and immediate engagement. Visual content allows for a deeper emotional connection with the audience, facilitating the analysis of the associations among visuals, aesthetics, consumer behavior, and PSRs. Furthermore, Instagram’s features such as stories, reels, and the ability to post longer captions enable a rich variety of PSI opportunities that are not as prevalent on other platforms. This provides a more comprehensive dataset for studying influencer-follower dynamics. Beauty and fashion influencers were chosen because these domains are highly visual, making Instagram’s image- and video-centric platform ideal matches. Based on the data from Statista, retail sales in Japan’s apparel industry reached approximately 8.1 trillion yen in 2022, whereas the market value of Japanese cosmetics shipments will be approximately 2.4 trillion yen [32,33]. Japanese fashion trends not only influence the Asian market, but also deeply influence the international fashion market. The beauty and fashion industry is a market with a large amount of available data. Japanese market and fashion and beauty influencers have significant research value. Previous research has paid little attention to this area, and this study provides a comprehensive understanding of the dynamics between fashion and beauty influencers and their followers in the Japanese market.

We examined the association between changes in the content categories, content value, and strength of PSIs and changes in PSR. Consequently, the dependent variable was defined as “PSRs’ strength changes.” The explanatory variables included “PSI changes,” “Value of influencer’s post changes,” and four categories (“PR changes,” “Clothing coordination changes,” “Beauty-related posts changes,” and “Style introduction changes”). We also considered the interactions between “each category’s increase/decrease” and “PSI increase/decrease,” as well as the interactions between “influencers’ posts value increase/decrease” and “PSI increase/decrease.” Each variable was analyzed statistically. The following sections provide an overview of the analytical strategy, the dataset and methods used to calculate the variables and present the specifications of the statistical analyses.

### 3.1 Analytical approach

We aimed to address the research question regarding the changes in PSRs’ strength, the dependent variable in the analysis, by comparing influencers’ posts from April 2021 with those six months later, in October 2021. Given the multitude of influencer domains on Instagram and the impracticality of including all domains in the data, we focused on influencers within a single domain: the beauty and fashion.

Research suggests that influencers can form PSRs with their followers through PSIs [13,39]. In turn, followers are influenced by the value of influencers’ posts, which are associated with PSRs [6,13,20,41]. Accordingly, we analyzed the association between changes in PSI between influencers and followers and the value of influencers’ posts with PSRs’ strength. We also examined the interaction effect of changes in the value of influencers’ posts and PSI on this relationship.

Influencers’ posts were grouped into the following categories: “PR” (posts that directly promote products), “Clothing coordination” (posts on coordinating clothes), “Beauty-related contents” (posts on makeup), “Style introduction” (detailed posts on the influencer’s fashion and lifestyle), and “Others” (although some posts might fit into multiple categories). Descriptions of the influencers’ post categories are presented in Table 2.

**Table 2:** Description of influencers’ various post categories.

Category	Description	Example
**PR posts**	Posts specifically created to promote a product or brand. Characterized by direct mentions of product names, prices, or specific promotional content, such as discounts and sales. The primary purpose is to convey the product’s features and appeal directly to followers, stimulating interest and purchase desire.	A detailed image of a skincare product with a caption highlighting its unique benefits and a call to action.
**Clothing coordination posts**	Posts crafted to showcase influencer’s outfits and styling. Aim to provide followers with fashion ideas and references for outfit coordinate. Typically include multiple images or videos that highlight different angles and aspects of the outfit, detailed descriptions of the clothing items, where to purchase them, and styling tips.	Multiple images of an outfit with details on each clothing item and tips on how to style them.
**Beauty-related posts**	Posts designed for sharing information and techniques related to makeup and skincare. Aim to provide followers with knowledge and advice about beauty, including tutorials, before and after pictures, or product reviews.	A tutorial on makeup application with before and after pictures, and detailed captions about the products used.
**Style introduction posts**	Posts that offer a detailed introduction to the influencer’s fashion styles and lifestyles. Aim to evoke empathy toward the influencer’s personality and lifestyle, and strengthen connections with followers.	A series of Instagram stories, which provide followers with a genuine insight into the influencer’s everyday life and style, showcase the influencer’s daily routines, favorite spots, and fashion choices.
**Others**	Posts that do not fit into the above categories. Used as a reference point in the regression analysis and not included in the model.	N/A

To analyze the interaction effects, we employed the following method. For each influencer, each explanatory variable’s values were compared between the April and October posts, and the changes were recorded. We then created four patterns of dummy variables by combining the PSI changes with the changes in other explanatory variables: “PSI decrease and explanatory variable X decrease,” “PSI decrease and explanatory variable X increase,” “PSI increase and explanatory variable X decrease,” and “PSI increase and explanatory variable X increase” (coded as 1 if applicable, and 0 otherwise). Notably, the “decrease and decrease” dummy variable served as the baseline and was excluded from the analysis. The subsequent analysis focused on evaluating the effect values within the remaining three patterns to ascertain the presence and nature of interaction effects.

**Fig. 1:**
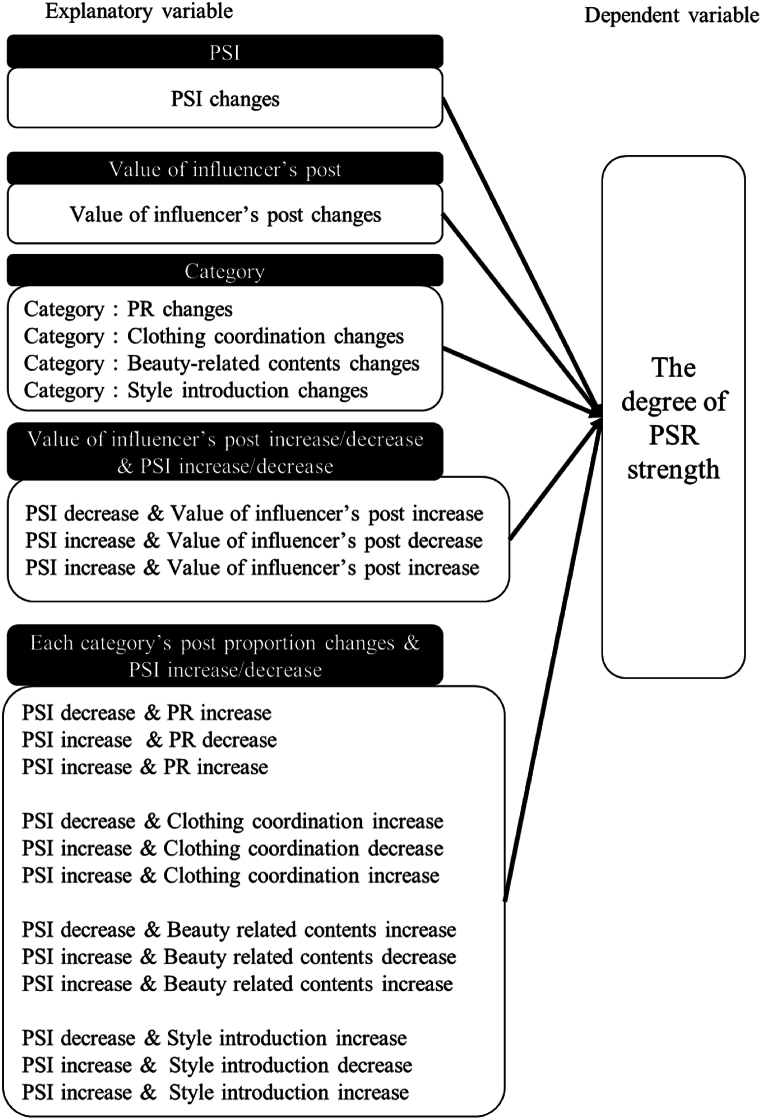
Analysis model.

Fig. 1 depicts a comprehensive visual representation of the analytical model used in this study. The individual influencer is the primary unit of analysis.

### 3.2 Data collection

#### 3.2.1 Instagram as an object of analysis

We utilized Instagram for data acquisition because its vast user base offers an abundance of data for analyzing the relationships between influencers and their followers [39]. Moreover, the platform provides numerous opportunities for PSI, such as followers leaving comments on influencers’ posts [61,62]. We collected data from influencers who posted during April and October 2021. These two months offer a view of influencer activity across different seasons. Social media marketing research suggests that even short-term interactions between users and influencers are sufficient to establish significant PSRs. Previous studies have demonstrated that a research cycle as short as nine weeks can effectively capture key marketing activities and user responses [63,64]. So we ensured a more comprehensive understanding of influencer-follower dynamics by selecting a six-month period that includes data from both spring and fall. Additionally, given Instagram's vast user base and its capacity for interactions, such as followers leaving comments on posts [61,62], the data collected during this period is representative and sufficient for our study’s objectives.

#### 3.2.2 Sampling

The data collection involved identifying influencers on Instagram. After initially identifying a single influencer, we used Instagram’s recommendation feature for “influencers similar to this influencer,” continuing until no new influencers were suggested. Once a comprehensive identification was completed, we filtered influencers who (1) posted about beauty and fashion, (2) posted during the two specific months of April and October 2021, and (3) had more than 1000 followers—a criterion based on Campbell and Farrell [65]. We collected data from 215 influencers.

A total of 7285 posts from 215 influencers were sampled between April and October 2021. The data collection period spanned from July 14, 2022, to September 29, 2022. However, influencers who set up accounts to keep their number of “likes” and comments private (such that the numbers were not visible to others) were excluded. This yielded 6678 posts from 167 influencers. We must emphasize the impact of this decision on the comprehensiveness of the dataset. Typically, more active and popular accounts tend to make their interaction data publicly available because they enhance their appeal and credibility [66]. Because our dataset includes only those influencers that make their interaction data publicly available, this selectivity bias may lead to an overrepresentation of highly active or popular influencers, while potentially ignoring influencers that are emerging or choose to adopt different strategies by keeping their interaction metrics private.

### 3.3 Variable description

Table 3 summarizes these variables. The specific details and calculation methods for each variable are thoroughly elucidated in the subsequent sections for comprehensive understanding.

**Table 3:** Summary of variables.

Variables	Description
Dependent variable	
$y_{i}^{O,PSR\;Strengths}$	Degree of change in PSR strength
Explanatory variables	
$x_{i}^{E,PSI}$	PSI changes
$x_{i}^{E,value}$	Value of influencer’s post changes
$x_{i}^{E,\text{contents},\mathrm{PR}}$	Changes in PR posts
$x_{i}^{E,\text{contents},\text{coordinate}}$	Changes in Clothing coordination posts
$x_{i}^{E,\text{contents},\text{beauty}}$	Changes in beauty-related content posts
$x_{i}^{E,\text{contents},\text{Style}\;\text{introduction}}$	Changes in style introduction posts
$x_{i}^{E,value,pattern,a}$	Interaction between a decrease in PSI and an increase in the value of an influencer’s post
$x_{i}^{E,value,pattern,b}$	Interaction between an increase in PSI and a decrease in the value of an influencer’s post
$x_{i}^{E,value,pattern,c}$	Interaction between an increase in PSI and an increase in the value of an influencer’s post
$x_{i}^{E,\mathrm{PR},pattern,a}$	Interaction between a decrease in PSI and an increase in PR
$x_{i}^{E,\mathrm{PR},pattern,b}$	Interaction between an increase in PSI and a decrease in PR
$x_{i}^{E,\mathrm{PR},pattern,c}$	Interaction between an increase in PSI and an increase in PR
$x_{i}^{E,\text{coordinate},pattern,a}$	Interaction between a decrease in PSI and an increase in clothing coordination
$x_{i}^{E,\text{coordinate},pattern,b}$	Interaction between an increase in PSI and a decrease in clothing coordination
$x_{i}^{E,\text{coordinate},pattern,c}$	Interaction between an increase in PSI and an increase in clothing coordination
$x_{i}^{E,\text{beauty},pattern,a}$	Interaction between a decrease in PSI and an increase in beauty-related content
$x_{i}^{E,\text{beauty},pattern,b}$	Interaction between an increase in PSI and a decrease in beauty-related content
$x_{i}^{E,\text{beauty},pattern,c}$	Interaction between an increase in PSI and an increase in beauty-related content
$x_{i}^{E,\text{Style}\;\text{introduction},pattern,a}$	Interaction between a decrease in PSI and an increase in style introduction
$x_{i}^{E,\text{Style}\;\text{introduction},pattern,b}$	Interaction between an increase in PSI and a decrease in style introduction
$x_{i}^{E,\text{Style}\;\text{introduction},pattern,c}$	Interaction between an increase in PSI and an increase in style introduction
Control variables	
$x_{i}^{C,followers}$	Number of followers
$x_{i}^{C,gender}$	Gender of the influencer
$x_{i}^{C,sector}$	Domain where the influencer is active

#### 3.3.1 Dependent variable

The dependent variable was the “degree of change in PSRs’ strength.” Building PSRs allows viewers to consider celebrities as intimate conversational partners [47]. Research shows that young people’s engagement with their favorite media personalities on Twitter (the name has been changed to X) and interactions and communication with them positively impact the strength of PSRs with these personalities [20]. Similarly, on Instagram, followers actively engage with influencers by leaving comments on their posts, which significantly contributes to developing these PSRs [39,67]. Commenting allows followers to establish interactive relationships with the influencers. Followers can express their opinions or ask questions through comments, to which influencers can respond in real time. This process facilitates the mutual sharing of knowledge and information, potentially deepening the followers’ interest and trust in the influencer. This strengthens PSRs, as reflected in the number of comments left by followers on influencer posts. Therefore, changes in comment count can be used to evaluate PSRs. Thus, we hypothesize that the number of followers’ comments on a post indicates the strength of the PSRs between influencers and followers.

We employed the following calculation method. (1) The number of comments by an influencer on each item was recorded in April and October 2021. (2) The average number of comments for each influencer was calculated. (3) The rate of change is calculated by dividing the average number of comments in October by those in April. (4) Initially observed to be highly skewed, the distribution was normalized by applying a natural logarithmic transformation to the rates and defining the transformed values as the variables $y_{i}^{O,PSR\;Strengths}$.

#### 3.3.2 Explanatory variables

We considered changes in the PSI, Value of influencer’s post, and post categories as the explanatory variables as follows: “PSI changes,” “Value of influencer’s post changes,” and the four post categories, “PR changes," “Clothing coordinate changes," “Beauty-related posts changes," and “Style introductions changes." Additionally, we included the interaction effects of “Value of influencer’s post changes” with “PSI changes,” and “changes in the posts in four specific categories” with “PSI.” Because the dependent variable represents a change, the explanatory variables were also calculated by comparing the values between April and October. We analyzed the relationship between these changes and the dependent variable, the number of comments (indicating the degree of PSRs’ strength).

To effectively analyze the interaction effects of each explanatory variable, changes were categorized into “increase” and “decrease” for each variable. This methodological approach was chosen over the direct multiplication of variables to calculate their interaction effects. The rationale was to mitigate the biases that could arise from significant differences among influencers, such as variations in follower counts. Direct multiplication may introduce distortions in the analysis due to these disparities, potentially leading to inaccurate interpretations of interaction effects.

Next, we described the calculation methods for these variables and explained the interaction effects of “Value of influencer’s post changes” with “PSI changes” and “each category’s post changes (‘PR changes,’ ‘Clothing coordination changes,’ ‘Beauty-related post changes,’ and ‘Style introductions changes’)” with “PSI changes.” These changes were calculated by comparing data from April to October. We found no instances where the data for April and October were identical. Therefore, the changes discussed here only involve increases or decreases, with no instances of change.

“PSI changes” were calculated by first determining the ratio of the number of times an influencer replied to follower comments in April and October 2021 to the total number of comments from followers in those months. The difference between the October and April results was used as an explanatory variable, denoted by $x_{i}^{E,PSI}$. To analyze the interaction effect of “PSI increase/decrease” on “Value of influencer’s post increase/decrease” and “each category’s (‘PR,’ ‘Clothing coordination,’ ‘Beauty-related post,’ and ‘Style introductions’) increase/decrease,” we distinguished between “PSI increase” and “PSI decrease.” This differentiation used the changes calculated in “PSI changes,” with a reference point of 0; values greater than 0 signify an “PSI increase,” whereas those less than 0 denote an “PSI decrease.” This distinction was made using values obtained before calculating the z-score.

“Value of influencer’s post changes” was calculated as follows: The value of an influencer’s post changes are determined by the number of likes, which reflect the perceived value of the influencer’s posts [20,41,61]. Thus, the number of likes that a post receives from its followers is assumed to indicate its value. Specifically, we computed the average number of likes for each influencer post in April and October 2021. This variable was calculated by taking the natural logarithm of the difference between these averages after converting them into z-scores. We termed this variable as $x_{i}^{E,value}$. To analyze the interaction effects of “PSI increase/decrease” and “Value of influencer’s post changes,” were categorized into a “Value of influencer’s post increases” and “Value of influencer’s post decreases.” The change derived from the value of the influencer’s post changes determined this categorization: values above zero were labeled as “Value of influencer’s post increase,” whereas values less than zero were labeled as “Value of influencer’s post decrease.” We then defined four interaction patterns based on combinations of “PSI increase/decrease” and “Value of influencer’s post increase/decrease.” For each pattern, the variable equals 1 if applicable and 0 otherwise. However, the pattern of “PSI decreases” and “Value of influencer’s post decrease” was used as a reference category in regression analysis and not included in the model. Table 4 presents the four interaction patterns along with the variables named for each.

**Table 4:** Four interaction patterns between “PSI increase/decrease” and “Value of influencer’s post increase/decrease”.

Pattern number	PSI	Value of influencer’s post	Variable representation in the model	Included in the model
(1)	Decrease	Decrease	Reference pattern in regression	No
(2)	Decrease	Increase	$x_{i}^{E,value,pattern,a}$	Yes
(3)	Increase	Decrease	$x_{i}^{E,value,pattern,b}$	Yes
(4)	Increase	Increase	$x_{i}^{E,value,pattern,c}$	Yes

“PR changes” are calculated as the z-score transformed difference in the proportion of direct product promotion posts by influencers between April and October, denoted by $x_{i}^{E,\text{contents},\mathrm{PR}}$. To further analyze the interaction effect, “PR changes” were divided into “PR increase” and “PR decrease.” Similar to the “Value of influencer’s post changes,” the changes calculated were used to create variables based on the four patterns of “PSI increase/decrease” and “PR increase/decrease.” For each pattern, the variable equals 1 if applicable and 0 otherwise. However, the “PSI decrease” and “PR decrease” patterns served as the reference category and were not included in the model. Table 5 presents the four interaction patterns along with the variables named for each.

**Table 5:** Four interaction patterns between “PSI increase/decrease” and “PR increase/decrease.”

Pattern number	PSI	PR	Variable representation in the model	Included in the model
(1)	Decrease	Decrease	Reference pattern in regression	No
(2)	Decrease	Increase	$x_{i}^{E,\mathrm{PR},pattern,a}$	Yes
(3)	Increase	Decrease	$x_{i}^{E,\mathrm{PR},pattern,b}$	Yes
(4)	Increase	Increase	$x_{i}^{E,\mathrm{PR},pattern,c}$	Yes

“Clothing coordination” was computed as the c hange in the proportion of influencer posts related to clothing coordination between April and October, transformed into a z-score, and represented by $x_{i}^{E,\text{contents},\text{coordinate}}$. The variable was categorized into “Clothing coordination increase” and “Clothing coordination decrease” depending on whether the change was positive or negative, respectively. For each pattern, the variable equals 1 if applicable and 0 otherwise. The “PSI decrease” and “Clothing coordination decrease” patterns were used as a reference in the regression analysis, and therefore, not included in the model. Table 6 presents the four interaction patterns along with the variables named for each.

**Table 6:** Four interaction patterns between “PSI increase/decrease” and “Clothing coordination increase/decrease.”

Pattern number	PSI	Clothing coordination	Variable representation in the model	Included in the model
(1)	Decrease	Decrease	Reference pattern in regression	No
(2)	Decrease	Increase	$x_{i}^{E,coordinate,pattern,a}$	Yes
(3)	Increase	Decrease	$x_{i}^{E,coordinate,pattern,b}$	Yes
(4)	Increase	Increase	$x_{i}^{E,coordinate,pattern,c}$	Yes

“Beauty-Related Posts” was calculated as the change in the proportion of beauty-related posts between April and October, transformed into a z-score, and designated as $x_{i}^{E,\text{contents},\text{beauty}}$. The variables were categorized as increases and decreases based on the direction of change. Each pattern was coded as 1 if it was present and 0 otherwise. Meanwhile, the “PSI decrease” and “beauty-related posts decrease” pattern was used as a reference in regression analysis, and hence, not included in the model. Table 7 presents the four interaction patterns along with the variables named for each.

**Table 7:** Four interaction patterns between “PSI increase/decrease” and “Beauty-related posts increase/decrease”.

Pattern number	PSI	Beauty-related posts	Variable representation in the model	Included in the model
(1)	Decrease	Decrease	Reference pattern in regression	No
(2)	Decrease	Increase	$x_{i}^{E,beauty,pattern,a}$	Yes
(3)	Increase	Decrease	$x_{i}^{E,beauty,pattern,b}$	Yes
(4)	Increase	Increase	$x_{i}^{E,beauty,pattern,c}$	Yes

“Style introduction” was computed as the change in the proportion of posts where an influencer introduces their fashion or lifestyle in detail, converted to a z-score, and denoted by $x_{i}^{E,\text{contents},\text{Style}\;\text{introduction}}$. The variables were categorized as increases and decreases based on the direction of change. Each pattern was coded as 1 if it was present and 0 otherwise. The “PSI decrease” and “Style introduction decrease” patterns served as a reference and were not included in the model. All categorizations were based on the original values before the z-score transformation. Table 8 presents the four interaction patterns along with the variables named for each.

**Table 8:** Four interaction patterns between “PSI increase/decrease” and “Style introduction increase/decrease.”

Pattern number	PSI	Style introduction	Variable representation in the model	Included in the model
(1)	Decrease	Decrease	Reference pattern in regression	No
(2)	Decrease	Increase	$x_{i}^{E,\text{Style}\;\text{introduction},pattern,a}$	Yes
(3)	Increase	Decrease	$x_{i}^{E,\text{Style}\;\text{introduction},pattern,b}$	Yes
(4)	Increase	Increase	$x_{i}^{E,\text{Style}\;\text{introduction},pattern,c}$	Yes

#### 3.3.3 Control variables

We considered other factors that could be associated with the strength of PSRs, such as the number of followers, cosmetics domain, apparel domain, and gender. Our choice of control variables was based on evidence from prior studies and the characteristics of our data. First, previous studies have widely established that gender may significantly affect PSR [68]. Therefore, based on this evidence, we used gender as a control variable. Second, we controlled for the number of followers. Credibility is an important indicator for establishing a strong PSR, and the number of fans is associated with the credibility of influencers [13]. Therefore, we include this as a control variable. Finally, we controlled for the clothing domain by considering the specific domains of data collection. As our data come primarily from influencers in both the beauty and apparel domains, there may be inherent biases in the data from different domains. While there are other potential influencers, such as influencers’ personalities and motivations, which may also be associated with PSR generation, given that this study relied on data collected by the platform, the impact of these individual differences may be treated as errors in the statistical analyses.

The variable “number of followers” was used as a measure of an influencer’s credibility and popularity. Influencers with fewer followers may have a strong influence within specific communities or niche areas, thereby enhancing the credibility of their followers. However, they are likely to have limited reach and do not command a broad range of influence. In contrast, influencers with numerous followers can reach many people because of their higher recognition and awareness. However, individual PSIs with followers tend to be limited [40,46]. Hence, the number of followers is associated with PSRs’ strength. We recorded the number of followers displayed on an influencer’s account at the time of data collection and converted it to a natural logarithmic form to create this variable; it was denoted by $x_{i}^{C,followers}$.

Influencers’ gender may also be associated with the perceived value of their posts and potentially have different associations with the strength of their PSRs with followers. Leung et al. [69] found significant gender differences in the associations of self-disclosure, prototype clarity, and self-prototypicality with influencers’ perceived trust and social attractiveness. Hence, sex was included as a control variable in our analysis. This ensured that the analysis accounted for potential differences in influencer effectiveness attributable to gender, thereby allowing for a more accurate assessment of the impact of other variables on the outcomes studied [69]. Influencers’ gender determination can be based on their posts up to the time of data collection. However, there were no cases of ambiguous sex within the scope of this study. Therefore, we defined a variable for the influencer’s gender, which was coded as 1 if the influencer was male and 0 otherwise. This variable is denoted by $x_{i}^{C,gender}$.

We focused on influencers in the cosmetics and apparel domains, recognizing that the specific elements of these sectors can be significantly associated with the strength of PSRs. Furthermore, given the unique consumer engagement and marketing strategies prevalent in these industries, we must distinguish between them. For example, the apparel domain involves frequent product launches and visual marketing [70]. This could be associated with the different strengths and characteristics of the PSI compared to those of the cosmetics domain. The cosmetics domain focuses on personal usage experience and detailed product demonstrations. Thus, a dummy variable for the apparel domain was established, coded 1 if the influencer was in the apparel sector and 0 otherwise. This variable is denoted by $x_{i}^{C,sector}$.

### 3.4 Specifications

We used multiple regression analysis to explore how various independent variables collectively influence a dependent variable. We conducted the analysis using the R programming language. We examined the data distribution and potential outliers by performing an exhaustive descriptive statistical analysis of the dataset. This includes calculating statistical indicators such as mean, standard deviation, maximum, minimum, and correlation between variables to ensure that the data meet the prerequisites for multiple regression analysis. Table 9 presents the descriptive statistics and correlations of the dependent, explanatory, and control variables. It can help identify potential multicollinearity issues and the strength and direction of the relationships among variables. Furthermore, histograms and Q-Q plots were used to assess the distribution of residuals, confirming that all models conformed to the normality assumptions. We ensured the reliability of our models by conducting the Durbin-Watson test for serial correlation and the Breusch-Pagan test for heteroscedasticity.

**Table 9:** Descriptive statistics and correlation tables.

	Mean value	Standard deviation	Maximum value	Minimum value	Psrs’ strength changes	Psi changes	Value of influencers Post changes	Category: pr changes	Category: clothing coordination Changes	Category: beauty-related content changes	Category: style introduction changes	Number of followers	Gender	Apparel domain
Psrs’ strength changes	0.00	1.00	1.13	−0.86	1.00									
Psi changes	0.00	1.00	3.40	−2.39	0.41	1.00								
Value of influencers post changes	0.00	1.00	1.15	−0.48	0.51	0.20	1.00							
Category: pr changes	0.00	1.00	4.57	−2.80	−0.01	−0.06	−0.09	1.00						
Category: clothing coordination changes	0.00	1.00	2.84	−3.23	0.09	0.08	0.17	−0.09	1.00					
Category: beauty-related content changes	0.00	1.00	4.65	−7.90	−0.11	0.06	0.01	−0.28	−0.18	1.00				
Category: style introduction changes	0.00	1.00	5.48	−3.78	−0.07	−0.08	−0.17	0.03	−0.25	−0.16	1.00			
Number of followers	0.00	1.00	2.57	−2.43	−0.02	0.04	−0.26	0.02	−0.18	0.11	−0.05	1.00		
Gender	0.59	0.49	1.00	0.00	−0.03	0.00	0.08	−0.06	0.10	0.09	−0.05	−0.38	1.00	
Apparel domain	0.92	0.27	1.00	0.00	0.00	−0.02	0.07	0.02	0.04	−0.12	−0.06	−0.32	0.31	1.00

The formulas for each analytical model and the details of the tests used to verify the reliability of the statistical models are described. *β* represents the coefficients that measure the effect of each predictor, ζ is the random error term, and *b* is the intercept of the model.

Model A: This model includes only control variables and serves as a baseline to assess the associations of control factors such as the number of followers, domain of expertise (cosmetics or apparel), and gender of the influencer with PSRs’ strength. The model can be expressed as follows:

$$y_{i}^{O,PSR\;Strengths}=\beta_{1}x_{i}^{C,followers}+\beta_{2}x_{i}^{C,gender}+\beta_{3}x_{i}^{C,sector}+\zeta_{i}+b\text{.}$$

Model B: Model B extends Model A by incorporating the explanatory variables: “PSI changes,” “Value of influencer’s post changes,” “PR changes,” “Clothing coordination changes,” “Beauty-related contents changes,” and “Style introduction changes.” This model assesses the direct association between these variables and the dependent variable without considering potential interaction effects. The model can be expressed as follows:

$$y_{i}^{O,PSR\;Strengths}=\beta_{1}x_{i}^{E,PSI}+\beta_{2}x_{i}^{E,value}+\beta_{3}x_{i}^{E,contents,\mathrm{PR}}+\beta_{4}x_{i}^{E,contents,coordinate}+\beta_{5}x_{i}^{E,contents,beauty}+\beta_{6}x_{i}^{E,contents,Style\;introduction}+\beta_{7}x_{i}^{C,followers}+\beta_{8}x_{i}^{C,gender}+\beta_{9}x_{i}^{C,sector}+\zeta_{i}+b\text{.}$$

Model C-1: This model builds upon Model B by adding dummy variables for the interaction effects of “Post value increase/decrease” and “PSI changes” increase/decrease. It examined whether the direction of change in this variable, combined with the direction of the PSI, correlated significantly with the dependent variable. The model can be expressed as follows:

$$y_{i}^{O,PSR\;Strengths}=\beta_{1}x_{i}^{E,PSI}+\beta_{2}x_{i}^{E,value}+\beta_{3}x_{i}^{E,contents,\mathrm{PR}}+\beta_{4}x_{i}^{E,contents,coordinate}+\beta_{5}x_{i}^{E,contents,beauty}+x_{i}^{E,contents,Style\;introduction}+\beta_{7}x_{i}^{E,value,pattern,a}+{\beta_{8}x_{i}^{E,value,pattern,b}+\beta_{9}x_{i}^{E,value,pattern,c}+\beta }_{10}x_{i}^{C,followers}+\beta_{11}x_{i}^{C,gender}+\beta_{12}x_{i}^{C,sector}+\zeta_{i}+b\text{.}$$

Model C-2: Similar to Model C-1, Model C-2 includes Model B and adds dummy variables for the interaction effects of “PR increase/decrease” and “PSI increase/decrease.” It analyzed whether the combined effect of changes in both PR-related posts and PSI correlated with PSR strength. The model can be expressed as follows:

$$y_{i}^{O,PSR\;Strengths}=\beta_{1}x_{i}^{E,PSI}+\beta_{2}x_{i}^{E,value}+\beta_{3}x_{i}^{E,contents,\mathrm{PR}}+\beta_{4}x_{i}^{E,contents,coordinate}+\beta_{5}x_{i}^{E,contents,beauty}+{\;\beta }_{6}x_{i}^{E,contents,Style\;introduction}+{\;\beta }_{7}x_{i}^{E,\mathrm{PR},pattern,a}+{\beta_{8}x_{i}^{E,\mathrm{PR},pattern,b}+{\;\beta }_{9}x_{i}^{E,\mathrm{PR},pattern,c}+\beta }_{10}x_{i}^{C,followers}+\beta_{11}x_{i}^{C,gender}+\beta_{12}x_{i}^{C,sector}+\zeta_{i}+b\text{.}$$

Model C-3: This model includes Model B and introduces dummy variables for “Clothing coordination increase/decrease” and “PSI increase/decrease,” exploring the interactive effect of changes in both clothing coordination-related content and PSI. The model can be expressed as follows:

$$y_{i}^{O,PSR\;Strengths}=\beta_{1}x_{i}^{E,PSI}+\beta_{2}x_{i}^{E,value}+\beta_{3}x_{i}^{E,\text{contents},\mathrm{PR}}+x_{i}^{E,\text{contents},\text{coordinate}}+\beta_{5}x_{i}^{E,\text{contents},\text{beauty}}+\beta_{6}x_{i}^{E,\text{contents},\text{Style}\;\text{introduction}}+\beta_{7}x_{i}^{E,\text{coordinate},pattern,a}+{\beta_{8}x_{i}^{E,\text{coordinate},pattern,b}+\beta_{9}x_{i}^{E,\text{coordinate},pattern,c}+\beta }_{10}x_{i}^{C,followers}+\beta_{11}x_{i}^{C,gender}+\beta_{12}x_{i}^{C,sector}+\zeta_{i}+b\text{.}$$

Model C-4: Following the same logic, Model C-4 adds to Model B dummy variables for “Beauty-related posts increase/decrease” and “PSI increase/decrease,” investigating how changes in both beauty-related content and PSI work together. The model can be expressed as follows:

$$y_{i}^{O,PSR\;Strengths}=\beta_{1}x_{i}^{E,PSI}+\beta_{2}x_{i}^{E,value}+\beta_{3}x_{i}^{E,contents,\mathrm{PR}}+\beta_{4}x_{i}^{E,contents,coordinate}+\beta_{5}x_{i}^{E,contents,beauty}+\beta_{6}x_{i}^{E,contents,Style\;introduction}+\beta_{7}x_{i}^{E,beauty,pattern,a}+{\beta_{8}x_{i}^{E,\text{beauty},pattern,b}+\beta_{9}x_{i}^{E,\text{beauty},pattern,c}+\beta }_{10}x_{i}^{C,followers}+\beta_{11}x_{i}^{C,gender}+\beta_{12}x_{i}^{C,sector}+\zeta_{i}+b\text{.}$$

Model C-5: Finally, Model C-5 includes Model B and incorporates dummy variables for “Style introduction increase/decrease” and “PSI increase/decrease,” examining the interaction effect of changes in both style introduction content and PSI. The model can be expressed as follows:

$$y_{i}^{O,PSR\;Strengths}=\beta_{1}x_{i}^{E,PSI}+\beta_{2}x_{i}^{E,value}+\beta_{3}x_{i}^{E,contents,\mathrm{PR}}+\beta_{4}x_{i}^{E,contents,coordinate}+\beta_{5}x_{i}^{E,contents,beauty}+\beta_{6}x_{i}^{E,contents,Style\;introduction}+\beta_{7}x_{i}^{E,Style\;introduction,pattern,a}+{\beta_{8}x_{i}^{E,Style\;introduction,pattern,b}+\beta_{9}x_{i}^{E,Style\;introduction,pattern,c}+\beta }_{10}x_{i}^{C,followers}+\beta_{11}x_{i}^{C,gender}+\beta_{12}x_{i}^{C,sector}+\zeta_{i}+b\text{.}$$

We confirmed that there were no multicollinearity concerns in any model, as shown by the variance inflation factor (VIF) values: Model A has an average VIF of 1.21 and a maximum of 1.23. Model B has an average VIF of 1.20 and a maximum VIF of 1.39. Models C-1, C-2, C-3, C-4, and C-5 have average VIFs of 1.82, 1.62, 1.83, 2.31, and 1.80, respectively, and maximum VIFs of 3.93, 2.72, 3.37, 6.49, and 3.15, respectively.

We assessed the distribution of residuals using histograms and Q-Q plots, which confirmed that non-normality was not an issue in any model. Thus, the residual distributions adequately conformed to the normality assumptions.

Serial correlations were examined using the Durbin–Watson test. As the null hypothesis of this test is “no serial correlation,” a p-value of ≥0.05 would indicate no serial correlation issues. However, as the p-values were less than 0.05, all the estimates were adjusted using the Newey–West method.

Finally, the Breusch–Pagan test was used to check for heteroscedasticity. The null hypothesis of the Breusch–Pagan test is “constant variance.” Therefore, a p-value ≥0.05 would suggest no heteroskedasticity issues. In Model A, the p-value was 0.01, requiring an adjustment of the estimates using the Newey–West method. For Models B, C-1, C-2, C-3, C-4, and C-5, the p-values were ≥0.05, confirming that heteroscedasticity was not a concern.

## 4 Results

Table 10 presents the results of the regression analysis. Initially, Model A incorporated control variables such as number of followers, apparel domain, and gender. The findings showed that these variables were not significantly associated with the strength of PSRs, as all p-values exceeded 0.05 (*p* > 0.05)

**Table 10:** Regression results.

	Model A	Model B	Model C-1	Model C-2	Model C-3	Model C-4	Model C-5
PSI changes		0.14∗∗ (0.04)	0.05 (0.04)	0.06 (0.04)	0.05 (0.04)	0.06 (0.04)	0.06 (0.04)
Value of influencer’s post changes		0.61∗∗ (0.08)	0.38∗∗ (0.10)	0.59∗∗ (0.08)	0.57∗∗ (0.09)	0.59∗∗ (0.09)	0.59∗∗ (0.08)
Category: PR changes		0.00 (0.02)	0.01 (0.02)	−0.02 (0.02)	0.00 (0.02)	0.01 (0.02)	0.01 (0.02)
Category: Clothing coordination changes		−0.01 (0.03)	0.00 (0.03)	−0.01 (0.03)	0.00 (0.04)	−0.01 (0.03)	−0.01 (0.03)
Category: Beauty-related content changes		−0.06 (0.03)	−0.05 (0.04)	−0.05 (0.03)	−0.05 (0.03)	−0.04 (0.03)	−0.05 (0.03)
Category: Style introduction changes		0.01 (0.02)	0.01 (0.02)	0.01 (0.02)	0.01 (0.02)	0.01 (0.02)	0.01 (0.02)

PSI decrease and value of influencer’s post increase			0.17∗ (0.07)				
PSI increase and value of influencer’s post decrease			0.18∗ (0.07)				
PSI increase and value of influencer’s post increase			0.37∗∗ (0.10)				

PSI decrease and PR increase				0.07 (0.07)			
PSI increase and PR decrease				0.17∗ (0.07)			
PSI increase and PR increase				0.26∗∗ (0.09)			

PSI decrease and Clothing coordination increase					−0.05 (0.08)		
PSI increase and Clothing coordination decrease					0.13† (0.08)		
PSI increase and Clothing coordination increase					0.20∗ (0.10)		

PSI decrease and beauty-related content increase						−0.06 (0.10)	
PSI increase and beauty-related content decrease						0.19 (0.12)	
PSI increase and beauty-related content increase						0.11 (0.09)	

PSI decrease and style introduction increase							0.00 (0.06)
PSI increase and style introduction decrease							0.16† (0.09)
PSI increase and style introduction increase							0.18∗ (0.08)

Number of followers	−0.01 (0.93)	0.04 (0.03)	0.04 (0.03)	0.03 (0.03)	0.03 (0.03)	0.04 (0.03)	0.04 (0.03)
Gender	−0.03 (0.69)	−0.01 (0.05)	−0.01 (0.06)	0.00 (0.05)	0.00 (0.05)	0.03 (0.07)	0.00 (0.05)
Apparel domain	0.00 (0.24)	−0.02 (0.06)	−0.03 (0.05)	−0.01 (0.06)	−0.02 (0.06)	−0.02 (0.06)	−0.02 (0.06)
Constant	0.05 (2.18)	0.01 (0.06)	−0.14∗ (0.06)	−0.09 (0.07)	−0.06 (0.11)	−0.02 (0.10)	−0.07 (0.10)
Adjusted R-squared	−0.17	0.36	0.40	0.37	0.37	0.37	0.37

∗∗*p* < 0.01, ∗*p* < 0.05, †*p* < 0.1.

Expanding on Model A, Model B included additional explanatory variables: “PSI changes,” “Value of influencer’s post changes,” and various content categories (“PR changes,” “Clothing coordination changes,” “Beauty-related posts changes,” and “Style introduction changes”). Model B results highlighted that “PSI changes” (*β* = 0.14, *p* < 0.001) and “Value of influencer’s post changes” (*β* = 0.61, *p* < 0.001) were significantly associated with PSR strength. However, changes in different category types had no significant relationship, indicating that not all category changes were equally associated with changes in the PSR.

**Fig. 2:**
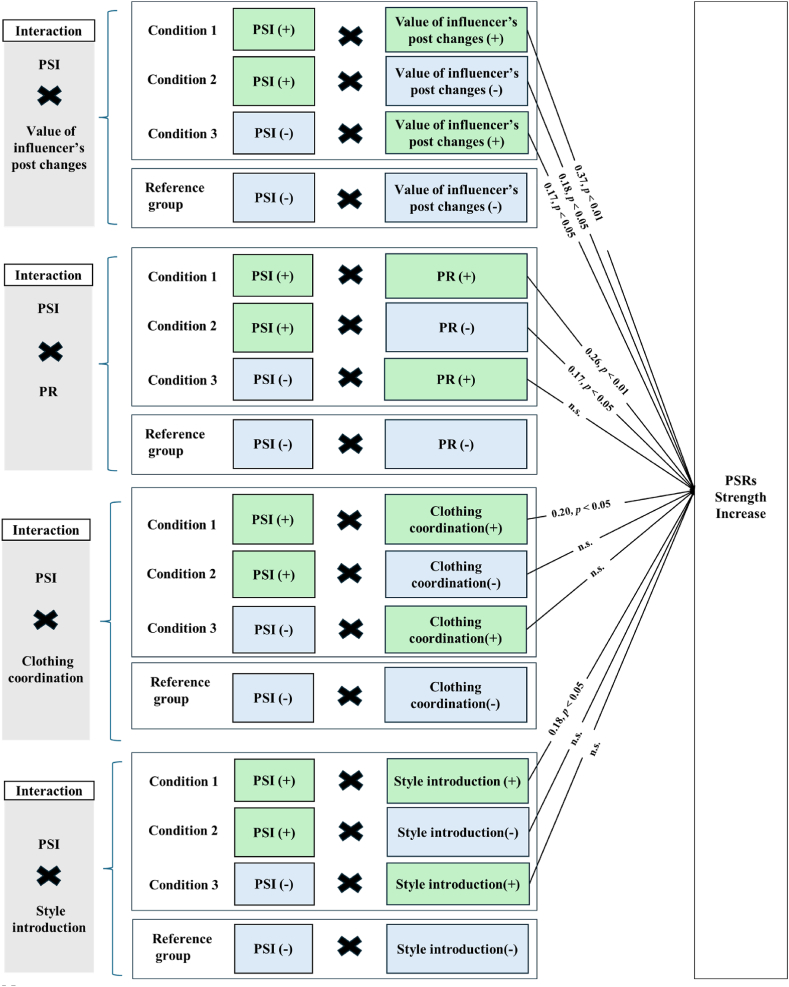
Result of interaction pattern. Note. 1. This figure is based on our results and illustrates the interactions for each category. However, for the beauty-related content category, because every increase or decrease pattern is irrelevant to the PSR, we have omitted it from the figure. 2. A “(+)” indicates an increase, while a “(−)” denotes a decrease. 3. p > 0.05 are labeled as “n.s.” to denote no statistical significance.

Model C examined interactions between “PSI changes” and four categories, “PR changes,” “Clothing coordinate changes,” “Beauty-related posts changes,” and “Style introductions changes,” compared to the reference group where both variables decreased. Fig. 2 shows a diagram created to better understand the interactions between each category and the PSI. However, for the beauty category, because every increase or decrease pattern is irrelevant to the PSR, we omitted it from the diagram. Model C-1 analyzed the combinations of “PSI changes” and “Value of influencer’s post changes,” revealing a significant correlation in several scenarios compared to the reference group. Specifically, both an increase in PSI coupled with a decrease in the value of the influencer’s post (*β* = 0.18, *p* < 0.05), and an increase in both PSI and post value (*β* = 0.37, *p* < 0.001) showed a significant positive correlation. Even a decrease in PSI combined with an increase in post value had a significant positive correlation (*β* = 0.17, *p* < 0.05). Thus, an increase in either PSI or the value of influencers’ posts can significantly correlate with the strength of PSRs. Model C-2 focused on “PSI changes” and “PR changes,” finding that “PSI increase,” whether combined with a “PR increase” (*β* = 0.17, *p* < 0.05) or a “PR decrease” (*β* = 0.26, *p* < 0.01), resulted in significant improvements in PSR strength compared to the reference group. Model C-3 addressed “PSI changes” and “Clothing coordination changes,” finding a significant positive correlation only when both increased (*β* = 0.20, *p* < 0.05) compared to the reference group (both deceased). Model C-4, which focused on “PSI changes” and “Beauty-related posts changes,” found no significant associations compared to the reference group. Lastly, Model C-5 focused on “PSI changes” and “Style introduction changes,” where increases in both were significantly associated with improved PSR strength (*β* = 0.18, *p* < 0.01) compared to the reference group.

In summary, strategic adjustments in “PSI changes” and “Value of influencer’s post changes” are significantly correlated with PSRs. Specifically, increasing PSI and enhancing content quality, particularly in “PR changes “and “Style introduction changes” and style introduction changes, are important in fostering stronger parasocial bonds between influencers and their audiences. This underscores the nuanced roles of various content changes and their interactions with the PSI in the dynamic landscape of social media influence. However, the effectiveness of these results may vary depending on cultural contexts.

## 5 Discussion

### 5.1 Interpretation of results and implications

#### 5.1.1 Association between PSI increase and PSR strength

The results for Model B demonstrate that an increase in PSI between influencers and followers is positively and significantly related to the strength of their PSRs. Thus, enhanced PSIs such as replying to comments are associated with stronger PSRs between influencers and their followers. Specifically, Bond [48] finds that PSIs on Twitter, including retweets and replies, were associated with stronger PSRs among young people. Rafaeli and Ariel [71] noted that these PSIs involve not only information exchange but also emotional engagement, which enhances closeness and understanding. Extending these findings, using data from the Japanese market, we demonstrate that increased PSIs deepen these connections.

#### 5.1.2 Association between the increase in value of influencer’s post and PSR strength

The results of Model B again illustrate that an increase in the value of the influencer’s post is significantly associated with stronger PSRs. Research has shown that “liking” posts on official Instagram accounts reflect the receiver’s reaction to those posts. By liking a post, the receiver ultimately develops an interest in following the influencer and builds a proactive relationship [[72], [73], [74]]. The value of influencers’ posts also correlates with the PSRs of followers [20,41], which is consistent with our findings. Furthermore, we analyzed the relationship between changes in the value of influencers’ posts and PSRs’ strength, suggesting that when influencers increase the value of their posts, their PSRs’ strength is enhanced. This may be because, when influencers provide attractive and engaging content that meets followers’ needs and demands, they may perceive these posts as beneficial, which is associated with stronger PSRs.

#### 5.1.3 Interaction effect of increases/decreases in both the value of influencer’s post and PSI on PSRs’ strength

According to Model C-1’s regression results, compared to the reference group (both decrease), either an increase in PSI or the value of influencers’ posts is associated with an increase in PSR strength. However, a comparison of their coefficients revealed that the correlation was significantly stronger when both increased simultaneously. Studies show that PSRs between influencers and followers are correlated with PSIs and the value of influencers’ posts [20,39,48]. We found that increases in both PSI and the value of the influencer’s post were significantly correlated with PSRs’ strength of PSRs. Additionally, when both simultaneously increase, we expect a stronger bond to be created between the influencer and followers, leading to stronger PSRs. This may be because an increase in PSI enhances communication and trust-building, prompting followers to develop more positive feelings toward the influencer. Simultaneously, as the value of the posts increases, followers receive more valuable content associated with stronger PSRs.

#### 5.1.4 Interaction effect of increases/decreases in both PR posts and PSI on PSRs’ strength

The results of Model C-2 showed no significant correlation when PSI decreased and PR-related posts increased. However, when PSI increased and the number of PR posts decreased, the PSR strength significantly increased. Furthermore, an increase in both PSI and PR posts has a stronger impact on PSR strength, with a coefficient of 0.26, indicating a more pronounced association. Boerman [75] observes that followers often react negatively to influencers who excessively promote products, potentially terminating their connections with those seen as overly commercial. Contrary to the general aversion toward high levels of promotional content, our findings suggest a nuanced perspective. Although an increase in PSI is positively correlated with PSRs, even when PR posts decrease, a simultaneous increase in both PSI and PR posts results in a more pronounced enhancement in PSR strength. This may be because although followers are aware of influencers’ promotional motives, they receive personalized feedback and answer questions during the PSI process. Additionally, while an increase in PR posts may initially be perceived negatively, if it is accompanied by an increase in PSI, this interaction not only helps followers better understand the advertised products but also emphasizes the influencer’s expertise as an opinion leader.

#### 5.1.5 Interaction effect of increases/decreases in both clothing coordination posts and PSI on PSRs’ strength

The results of Model C-3 show that an increase in both the PSI and clothing coordination posts has a significant positive correlation with the strength of PSRs. Followers check photos of influencers wearing coordinated products and understand the influencer’s fashion style, which helps build PSRs [6,34,76,77]. Consequently, followers may mimic the influencers’ behavior and appearance [40,78]. We demonstrate that an increase in clothing coordination posts, along with an increased PSI between influencers and followers, is associated with a positive enhancement in PSRs. One reason for this may be that clothing coordination posts tend to provide one-sided information from the influencer, focusing on providing information rather than fostering empathy or sharing interests with followers. Conversely, a PSI with an influencer emphasizes shared interests in fashion, allowing followers to gain opinions, ideas, and inspiration related to their style. This helps to deepen their connections with the influencer. Consequently, followers are more likely to actively participate, thereby increasing their empathy for and trust in influencers.

#### 5.1.6 Interaction effect of increases/decreases in both beauty-related posts and PSI on PSRs’ strength

Model C-4 reveals that the three variables—PSI decrease and beauty-related posts increase, PSI increase and beauty-related posts decrease, and PSI increase and beauty-related posts increase—have no significant correlations. Followers’ limited sensitivity to changes in beauty-related posts suggests that their engagement with an influencer may be deeply rooted in the influencer’s overall persona. This implies that followers may form attachments based on the influencer’s personality, style, or consistency of character, which transcends the details of individual makeup posts. Therefore, the increase or decrease in the effectiveness of posts in the beauty category may be secondary to how well these changes align with the influencer’s established persona and expectations set by their typical content narrative [65].

#### 5.1.7 Interaction effect of increases/decreases in style introduction posts and PSI on PSRs’ strength

The results of Model C-5 showed that an increase in both PSI and style introduction posts significantly enhanced PSRs’ strength. Style introduction posts focus on the influencers’ individuality and lifestyle. Influencers can increase the PSIs in their accounts by recommending products that fit their style [39]. Conde and Casais [40] contend that influencers can build intimate relationships with followers by showcasing their personal lives. Our results indicate that an increase in style introduction posts accompanied by an increase in PSI significantly enhances PSRs’ strength. This may be because followers are interested in the influencer’s style and, through PSIs, seek style advice suited to them, thus highlighting the influencer’s importance to followers and fostering a deeper interest. This closeness can lead to greater trust in the influencer, which is associated with stronger PSRs.

### 5.2 Theoretical implications

As mentioned in the introduction, the rapid growth of social media influencer marketing research aligns with the expansion of highly interactive and visual content-focused social media platforms [28]. Additionally, PSI and PSR are relatively new and prominent themes in social media influencer marketing [28]. However, academic research has not fully explored how to quantify the value of content, its categories, and their correlations with PSR. Furthermore, gaining deeper insight into what makes PSR satisfying and enduring can significantly enhance our understanding of these relationships. Our study addresses these key gaps in the literature by demonstrating how the value of content and the dynamic interactions between content categories and PSI correlate with the strength of PSRs, thus revealing the interplay between these variables. Our findings show that the content value and category of content are significantly correlated with PSR. This highlights the importance of content management in maintaining and enhancing PSR, contributing to social media influencer marketing theory. In addition, our study extends the theoretical framework by incorporating the concepts of content value and categorization into the PSI theory. This provides a basis for constructing a more comprehensive model that articulates how influencers can incorporate PSI to promote PSR. The following three paragraphs describe how this study addresses these gaps: The first section discusses the correlation between content value and PSR; the second discusses the association between different content categories and PSR; and the last paragraph focuses specifically on changes in the PSR relationship in practice, discusses how changes in content categories shape the deployment of PSI, which in turn affects the development of PSR.

First, we demonstrate a significant correlation between the value of an influencer’s post and PSR strength. Using the number of likes to represent the value of a post, we found that high-value posts, which attract consumer attention are more effective in promoting PSRs. Research indicates that consumer-brand resonance helps facilitate the relationship between consumers and brands [31]. We build on this foundation by clarifying that the value of influencers’ posts, as measured by consumer favorites, improves the PSR between influencers and attentive facilitators. This is consistent with existing research highlighting that consumers seek personalized and engaging influencer content that resonates deeply with them, thereby fostering PSRs with influencers [31].

Second, our study finds that different content categories have different correlations with PSR strength, suggesting that the role of content in influencer marketing strategies is more complex and subtle than previously recognized. Our findings extend the work of Cheung et al. [22], who examined various characteristics of influencer post content such as information quality, design, technology, and creativity, but did not specifically focus on the implications of content categorization for PSRs. Effective categorization helps users identify relevant products and supports their decision-making processes, thereby improving their market understanding and performance [27]. For example, the differential correlation between beauty-related content and clothing-coordinated posts on PSR strength suggests that followers are not uniformly sensitive to changes in content, but respond in a contextually relevant manner. This finding challenges the existing one-size-fits-all approach to marketing media influencer marketing [79]. This creates opportunities to explore the specific conditions under which certain types of content are more effective in promoting PSR.

Third, in the case of PSR in social media influencer marketing, understanding what makes PSR satisfying and enduring can contribute to a better comprehension of PSRs and how social media influencers can cultivate and sustain these relationships over time. Our study addresses key gaps in the existing literature by demonstrating how content value, content category, and the dynamic interactions between them, and PSI, correlate with PSR strength. We find that the strength of PSI’s influence on PSR varies significantly with changes in content categories. Thus, not all influencer-follower PSIs enhance PSRs equally; their effectiveness depends heavily on how the content is strategically categorized and presented. We found that while an increase in PSI is associated with stronger PSRs when PR posts decrease, a simultaneous increase in both PSI and PR posts increases PSR strength. Studies have demonstrated that excessive advertising, often perceived negatively by consumers, can undermine PSRs [[52], [53], [54]]. Our findings indicate that the strategic management of PSIs and PR can counteract consumers’ negative perceptions of advertisements. Thus, an increase in PSIs in conjunction with the content of PR releases enables influencers to reconcile consumer skepticism and enhance the overall effectiveness of marketing strategies. Additionally, we demonstrate that an increase in clothing coordination posts, along with an increased PSI between influencers and followers, is associated with a positive enhancement of PSRs. We also demonstrate that an increase in style introduction posts, along with increased PSI between influencers and followers, is associated with the positive enhancement of PSRs. However, for beauty-related content, changes in both PSIs and the number of beauty-related posts had no significant impact on the PSRs. Followers’ sensitivity to changes in beauty-related posts is limited, indicating that their interactions with influencers may be deeply rooted in the latter’s overall image. Overall, by analyzing the effects of category-specific content, our study deepens the understanding of the complex dynamics influencing PSRs on social media platforms and provides valuable insights into the strategic presentation of content categories and their varied impacts on these relationships.

### 5.3 Practical implications

Influencer marketing is growing rapidly [6,13,80], with influencers typically interacting with consumers through various platforms and methods, and providing information and advice about products to realize their business value. Based on our findings, we highlight the following implications for companies considering influencer marketing.

First, we introduce new criteria for selecting influencers for companies that use influencers on visually oriented platforms, such as Instagram. Higher PSR strength suggests greater trust in influencers among followers. This can be positively associated with the consumers’ influencer-promoted product reception. Therefore, when developing influencer marketing strategies, companies must consider increasing or decreasing PSIs between influencers and followers to gauge the strength of their PSRs. Selecting influencers who interact actively with their followers can lead to more reliable marketing strategies.

**Fig. 3:**
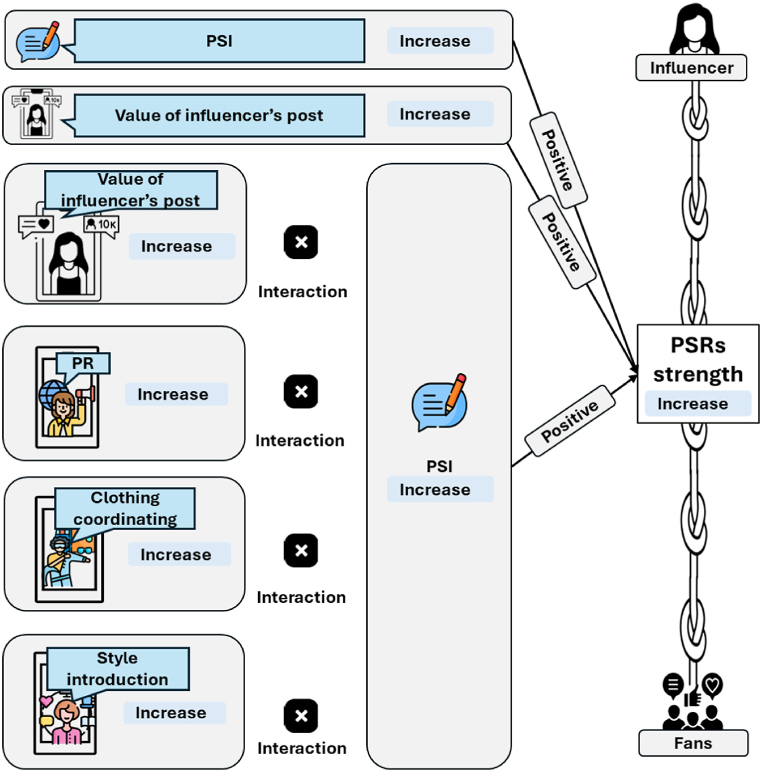
Implications for enhancing PSR strength.

Next, this study provides new approaches for fostering PSRs between influencers and followers, as illustrated in Fig. 3. Because an increase in the value of influencers’ posts positively influences PSRs’ strength, influencers can build trust with their followers by providing high-value posts that meet their needs and interests. The interaction effects of PSI increase/decrease and changes in each category’s proportion of posts suggest that increasing posts in categories such as PR and clothing coordination, combined with increased PSI, is desirable. This creates emotional resonance and intimacy with followers, thereby enhancing their PSR strength. We find that a PSI increase, coupled with a strategic focus on specific content categories like “PR change” and “Clothing coordination changes,” is significantly associated with PSRs. This insight can guide influencers and brands in the beauty and fashion industries, effectively tailoring their engagement tactics. By prioritizing these content categories and fostering more frequent and meaningful PSIs, influencers can deepen their connections with their followers and resonate more effectively with their audiences. This approach optimizes digital reach and engagement and provides a practical framework for brands to enhance their marketing strategies. Overall, these findings offer actionable guidance to improve influencers’ marketing engagement.

### 5.4 Limitations and future research directions

We identified several areas that warrant further investigation to deepen our understanding of PSIs. First, this study focused on influencers in the fashion and beauty categories. Given the differences in expectations and engagement patterns of audiences in different categories, further validation of the applicability of these findings to influencers in other categories such as entertainment or sports is needed.

Second, although PSRs are often viewed positively and researchers actively explore ways to enhance them, we must recognize their limitations. Country and cultural differences, for example, may influence the difficulty of establishing and enhancing PSR, as audiences from different cultures may perceive and accept influencers differently [81,82]. This study focuses on specific social media platforms and influencers in Japan and provides insights into the country’s unique social media dynamics. However, these insights may not capture the full spectrum of global social media environments, where user interactions and engagements vary across cultural backgrounds. For example, Japan’s group-oriented culture shapes user interactions differently from more individualistic societies such as the United States and Australia, where self-expression and personal identity dominate social media usage [83]. Siva et al. (2023) found that in collectivism, influencers’ posts emphasize emotional competence and are designed to be interactive, whereas in individualism, influencers focus on informational competence self-promotion [84]. Therefore, we hypothesized that in individualistic cultures, the moderating role of PSI between categories and PSR may diminish. Conversely, the importance of the informational value delivered by the content and the promotional efforts of the influencer is likely to increase significantly. Future research should further validate this hypothesis.

Another significant limitation is our exclusive reliance on quantitative analysis, which may not fully capture PSIs’ intricate nuances. Considering that our dataset includes only those influencers who make their interaction data publicly available, this selective bias may lead to an overrepresentation of highly active or popular influencers, while potentially ignoring influencers who are emerging or who choose to adopt different strategies by keeping their “Likes” and “Comments” private. This may favor one model of influencer behavior and ignore an important group of people in the social media domain who use privacy as part of their interaction strategies. Therefore, we suggest that future research explore the diverse strategies employed by influencers who are less visible or those adopting different engagement strategies, to offer a more comprehensive and diverse perspective. Future studies should integrate qualitative methods such as in-depth interviews or focus groups with followers. The mixed-methods approach can provide a more nuanced understanding of how followers perceive and interact with influencers, and offer a more comprehensive view of the complexities involved in these relationships.

Furthermore, our focus on influencers in the beauty and fashion sectors limits the generalizability of our findings to other industries. Future research should explore influencers from diverse domains, focusing on how industry-specific characteristics and consumer preferences relate to PSIs. This may provide insight into the adaptability and effectiveness of influencer marketing strategies across sectors. In addition to the fixed characteristics of our focus industry, other potential correlating factors such as trust, fairness, and the personalities and motivations of influencers may also be associated with PSR generation. We suggest that future studies explore these additional factors. Moreover, the psychological and behavioral changes of followers will also lead them to react differently to the same behaviors of the influencers, and future studies should focus on the differences in individual followers. In addition, it would be interesting to incorporate the role of emerging trends (Augmented Reality, Virtual Reality use) in PSR. Future research could explore this aspect.

Finally, studies should differentiate and analyze the associations between positive and negative comments and their relationships. Examining areas prone to negative comments will help elucidate the nature of these PSIs and their correlations with the strength and quality of PSRs. By addressing these gaps, future research could build on the foundational findings of this study to enhance the effectiveness of digital marketing strategies in an increasingly interconnected social media landscape.

## 6 Conclusion

We explored the evolution of PSRs between influencers and their followers in the beauty and fashion domains on Instagram by analyzing data from 7285 posts by 215 influencers. Our analysis reveals associations between the value of the influencer’s posts, post categories, and the levels of PSI in these relationships.

Specifically, enhanced PSI between influencers and their followers is significantly associated with increased PSR strength. Influencers can resonate deeply with their followers by directly interacting with their comments. Moreover, the value of the influencer’s posts plays an important role; higher-value content is associated not only with more engagement but also with stronger relationships. This emotional resonance is reinforced when influencers consistently post content aligned with their followers’ needs and interests. This resonance is vital, as it taps into followers’ sense of identity and integrates influencer posts into their daily social media PSIs [31]. Notably, our findings indicate that an interaction effect arises when the value of the influencer’s post and PSI increase simultaneously, significantly boosting the strength of the PSRs. Furthermore, intensified PSIs, coupled with an increase in “PR,” “Clothing coordination, “and “Style introduction” posts, are significantly associated with greater PSR strength. Thus, by strategically selecting content categories and increasing PSIs, influencers can establish stronger connections, resonate with their audiences, and significantly enhance their PSR strength.

In conclusion, we demonstrate the interaction between post categories, influencers' post values, and PSI levels, which together shape PSR on social media platforms. At the theoretical level, this study extends the theoretical framework by incorporating the concepts of content value and categorization into the theory of PSIs. This lays the foundation for constructing a more comprehensive model that explains how influencers utilize PSI to promote PSRs. At the practical application level, our research suggests that when developing influencer marketing strategies, companies must consider increasing or decreasing PSI between influencers and followers to measure the strength of their prosocial relationships. By prioritizing certain content categories and facilitating more frequent and meaningful PSI, influencers can deepen their connection with followers and more effectively empathize with their audiences.

Despite some limitations, such as focusing only on Japanese influencers and relying exclusively on quantitative analysis, our findings provide valuable insights into the dynamics of PSI and PSR. Addressing these limitations in future research will help deepen the understanding and improve the effectiveness of influencer marketing across cultures and industries. This study fills an important gap in the literature by examining how content value, category, and PSI influence the dynamics of PSR in the fashion and beauty industries. It opens opportunities for future research to examine influencer-follower interactions in various industries and cultures, offering clearer strategies to strengthen PSRs and increase audience engagement.

## CRediT authorship contribution statement

**Xiaoxiao Zhou:** Writing – review & editing, Writing – original draft, Visualization, Validation, Software, Methodology, Formal analysis, Data curation, Conceptualization. **Yi Huang:** Writing – review & editing, Writing – original draft, Visualization, Validation, Supervision, Software, Resources, Methodology, Formal analysis, Data curation, Conceptualization. **Yuki Inoue:** Writing – review & editing, Writing – original draft, Visualization, Validation, Supervision, Software, Project administration, Methodology, Investigation, Funding acquisition, Formal analysis, Data curation, Conceptualization.

## Data availability statement

The data associated with this study have not been deposited in a publicly available repository. Data presented here are available upon request from the corresponding author.

## Funding

This work was supported by JSPS KAKENHI (grant no. 24K00287).

## Declaration of competing interest

The authors declare that they have no known competing financial interests or personal relationships that could have appeared to influence the work reported in this paper.

## Abbreviations:

PSR: Parasocial relationship
PSI: Parasocial interaction
SNS: Social networking sites
